# Glycogen synthase kinase 3β inhibitors prevent hepatitis C virus release/assembly through perturbation of lipid metabolism

**DOI:** 10.1038/s41598-017-02648-6

**Published:** 2017-05-31

**Authors:** Mohammed A. Sarhan, Mohamed S. Abdel-Hakeem, Andrew L. Mason, D. Lorne Tyrrell, Michael Houghton

**Affiliations:** 1 Department of Medical Microbiology and Immunology, University of Alberta, Edmonton, Alberta Canada; 2 Li Ka Shing Institute of Virology, University of Alberta, Edmonton, Alberta Canada; 3 Department of Microbiology and Immunology, National Liver Institute, Menoufia University, Menoufia, Egypt; 4 Department of Medicine, University of Alberta, Edmonton, Alberta Canada; 5 Department of Microbiology and Immunology, Faculty of Pharmacy, Cairo University, Cairo, Egypt

## Abstract

Direct acting antivirals against hepatitis C virus (HCV) have markedly improved cure rates in the past few years. However, they are expensive, with only few targeting host cell factors, and affecting virus assembly and release. Huh7.5 cells infected with a JFH-1 clone of HCV were treated with two different glycogen synthase kinase (GSK3)-β inhibitors; AR-A014418 and lithium chloride. Intra- and extracellular HCV virions and specific infectivity was determined using real-time RT-PCR and TCID50, and changes in lipid production were identified by enzyme-linked immunoassay and mass spectrometry analyses. Similarly, effect on two HCV replicon cells were identified by the luciferase activity. Although there was limited effect on virus replication in Huh7.5 cells and replicons, Huh7.5 cells treated with GSK3β inhibitors produced significantly less viral particles in comparison to untreated cells. In addition, the treated cells synthesized significantly lower amounts of ApoB and trapped the ApoE lipoproteins in the cells. In conclusion, our study suggests that GSK3β plays a pivotal role in HCV virion assembly and release mediated in part through inhibition of apolipoprotein synthesis.

## Introduction

Chronic hepatitis C virus (HCV) infection affects more than 170 million people worldwide, and accounts for about 66% of cirrhosis and hepatocellular carcinoma in the developed world. HCV infection is the main indication for liver transplantation in the United States^1^. The recent innovation of combination direct acting antivirals (DAAs) has resulted in a marked increase in sustained virological response rates in patients with chronic HCV infection. However, these drugs are very expensive and are therefore of limited access to the HCV patient populations around the world, so there is an urgent need for newer and cheaper HCV antivirals.

Targeting host cell proteins that are essential for HCV replication represents an alternative strategy to design antivirals with a broader spectrum and a higher barrier to resistance. Cyclophilin^2^, phosphatidylinositol 4-kinase alpha^3^, and heat shock proteins^4^ are all host proteins that have been targeted to inhibit HCV infection in cultured cells, and were also shown to reduce HCV viremia in patients^2^. Characterizing other host proteins involved in the different stages of HCV life cycle would potentially identify novel therapeutic targets for pan-genotypic antivirals with minimal resistance.

Glycogen synthase kinase (GSK)3 is a serine/threonine kinase that modulates multiple cellular pathways. GSK-3 is regulated by phosphorylation of the serine residues, which inactivates the protein^5–7^. GSK-3 is comprised of two highly conserved kinases, GSK-3α and GSK-3β, that are ubiquitously expressed but have different functions. Increasing interest in modulating GSK3 activity for treatment of diverse neoplastic, metabolic and neurological disorders has resulted in the development of different GSK3-specific inhibitors^8^.

GSK3β is a member of the cyclin-dependent kinase family that are thought to be involved in the phosphorylation of HCV non-structural protein 5 A (NS5A)^9^. The multifunctional NS5A protein lacks enzymatic activity but plays a key role in HCV virion production^9^. The pathway of assembly and secretion of HCV are closely linked with the metabolism of very low-density lipoproteins (VLDL) and apolipoproteins (Apo) E and B^9, 10^. While the role that GSK3 plays in other disorders has been studied in detail^8^, little is known about the potential interaction of GSK3β with HCV proteins impacting virion maturation and release or how GSK3β influences the lipid pathway.

In this study, we examined the regulatory effect of GSK3β on HCV replication and virion production in the human hepatoma cell line (Huh7.5), and the potential interaction with VLDL assembly and Apo E and B. We used two GSK3 inhibitors: lithium (Li), a non-selective GSK3 inhibitor and an FDA-approved mood stabilizer, and AR-A014418 (AR), a small molecule heterocyclic thiazole compound that acts as a selective GSK3β inhibitor. AR was developed by AstraZeneca and subsequently reported to inhibit tau phosphorylation and neuronal death in Alzheimer’s disease^11^. Using Li and AR, we found that GSK3β inhibition significantly inhibited the assembly/release of HCV viral particles from cells possibly due to inhibition of VLDL assembly.

## Methods

### Compounds

GSK3β inhibitors AR-A014418 and lithium chloride were purchased from Sigma-Aldrich (St. Louis, MO, USA).

### Cell culture and JFH-1 infection

Tissue culture adapted (TCA) JFH-1 (obtained from Dr. Lorne Tyrrell) was propagated in Huh-7.5 cells as previously reported^12, 13^. Briefly, cells were transfected with RNA transcribed from linearized DNA plasmids using the DMRIE-C transfection reagent (Invitrogen, Carlsbad, NM, USA). The cells were washed and cultured in Dulbecco’s modified Eagle’s medium (DMEM) for 72 h. The culture supernatants were assayed for infectious HCV titer by limiting dilution in a focus-formation assay. Titers were expressed as the TCID_50_ and calculated as previously reported^13, 14^. Primary human hepatocytes used as a control for expression of ApoE and ApoB were obtained from Dr. Lorne Tyrrell laboratory.

Cells were pretreated with GSK3β inhibitors for 24 h, then, infected with TCA JFH-1. After 4 h of infection, cells were washed to remove virus and placed in fresh DMEM with 10% fetal bovine serum/penicillin/streptomycin while maintaining GSK3β inhibitor treatment. RNA was isolated from cells and supernatants for quantification of HCV and TCA JFH-1 copy number (virus genome equivalent) by the real-time polymerase chain reaction (RT-PCR) as described previously^15^. Huh7.5 cells carrying the HCV subgenomic replicon (obtained from Dr. Ralf Bartenschlager) were maintained in DMEM containing 10% fetal bovine serum and 500 mg/mL G418. Cells were cultured in 96-well plates and treated with serial dilutions of Li or AR. Cells were examined after 48 h for luciferase expression.

### Immunohistochemistry

Fixed cells were examined by immunohistochemistry. In brief, Huh7.5 cells were stained in 96-well plates using mouse anti-NS5A monoclonal antibodies (EMD Millipore, Billerica, MA, USA) and examined by laser scanning cytometry (CompuCyte, Westwood, MA, USA) to measure the expression of the NS5A protein.

### Quantification of viral RNA and RT-PCR

For analysis of intracellular transcript levels, total RNA was extracted from cells using Trizol (Invitrogen). cDNA was synthesized using random primers and superscript II (Invitrogen) according to the manufacturer’s specifications. Quantitative RT-PCR was performed using a SYBR green super mix (Sofast, Bio-Rad, Hercules, CA, USA). The following primer pairs were used for PCR amplification of the HCV 5′-untranslated region (UTR): 5′TCT GCG GAA CCG GTG AGT A (sense; UTR1) and 5′GTG TTT CTT TTG GTT TTT CTT TGA GGT TTA GG (antisense; RTU1). Reaction mixtures were amplified for 40 cycles in a CFX96 RT-PCR machine thermocycler (Bio-Rad) by using the following conditions: denaturation at 95 °C for 1 min, annealing at 60 °C for 1 min, and extension at 72 °C for 1 min. Cycle threshold values were corrected for the specific PCR efficiency of the primer, and normalized to hypoxanthine phosphoribosyltransferase 1 transcript levels. The melting temperature was adjusted to avoid quantification of nonspecific primer dimers and to enhance product specificity.

### Laser scanning cytometry

Huh7.5 cells treated with GSK3β inhibitors or DMSO were cultured on 48-well plates infected and incubated following the same protocol as described above. Cells were permeabilized in 0.1% Triton X-100 for 15 min and stained with anti-NS5A monoclonal antibodies (EMD Millipore, Billerica, MA, USA). Plates were scanned for NS5A expression by laser scanning microscopy using the machine phantom integral calculation method. Non-infected cells were used as a control for the background.

### Electron microscopy

Huh7.5 cells treated with GSK3β inhibitors or DMSO were grown on Thermanox coverslips (Thermo Fisher Scientific, USA), fixed in 4% paraformaldehyde for 30 min at room temperature and then washed with phosphate-buffered saline. Using transmission electron microscopy, images were taken for Huh7.5 cells cultured with TCA JFH-1 using non-infected Huh7.5 cells as negative controls. About 10 sections were examined per treatment.

### Cell apoptosis and MTT assays

Cell viability was investigated in treated and non-treated cells following staining with Annexin V and propidium iodide (Sigma-Aldrich). Stained cells were examined by flow cytometry and data are presented as dot plots. Cell viability was also assessed with the (4,5-dimethylthiazol-2-yl)-2,5-diphenyltetrazolium bromide (MTT) assay. The intracellular purple formazan was solubilized and quantified by spectrophotometry at 570 nm.

### Western blotting and ELISA

Proteins were isolated from cells and treated with ice cold RIPA buffer (Sigma-Aldrich) as previously described^16^. Blots were then incubated with ApoB or ApoE monoclonal antibodies purchased from Genetex (Irvine, CA, USA) or β-catenin (eBioscience, San Diego, CA, USA). GSK3β phosphorylation was evaluated using a GSK3β Total/Phospho InstantOne ELISA (eBiosicience).

### Quantitation of albumin and human α1 antitrypsin secretion (hAAT)

The amount of secreted albumin was determined using an ELISA as described previously^17^. Albumin secretion was calculated as ng albumin/hour/10^7^ cells and expressed as the fold-increase compared to fetal bovine serum. hAAT was measured in 20 µL of tissue culture supernatant using the ELISA technique as described^17^.

### Fast protein liquid chromatography (FPLC) analysis of secreted lipoproteins

Lipoprotein analysis was done by the lipid core facility, utilizing concentrated cell culture supernatants and size-exclusion FPLC (large particles elute first) combined with in-line triglyceride and cholesterol measurements.

### Statistical analyses

Results were analyzed by a one-way analysis of variance (ANOVA) or unpaired Student t test with Welch’s correction using GraphPad Prism software (GraphPad Software, San Diego, CA, USA). Differences between experimental conditions were considered significant when two-sided P values were below or equal to 0.05, unless otherwise mentioned.

## Results

### GSK3β inhibitors increase phosphorylated GSK3β and decrease the degradation of β-catenin in Huh7.5 cells

**Figure 1:**
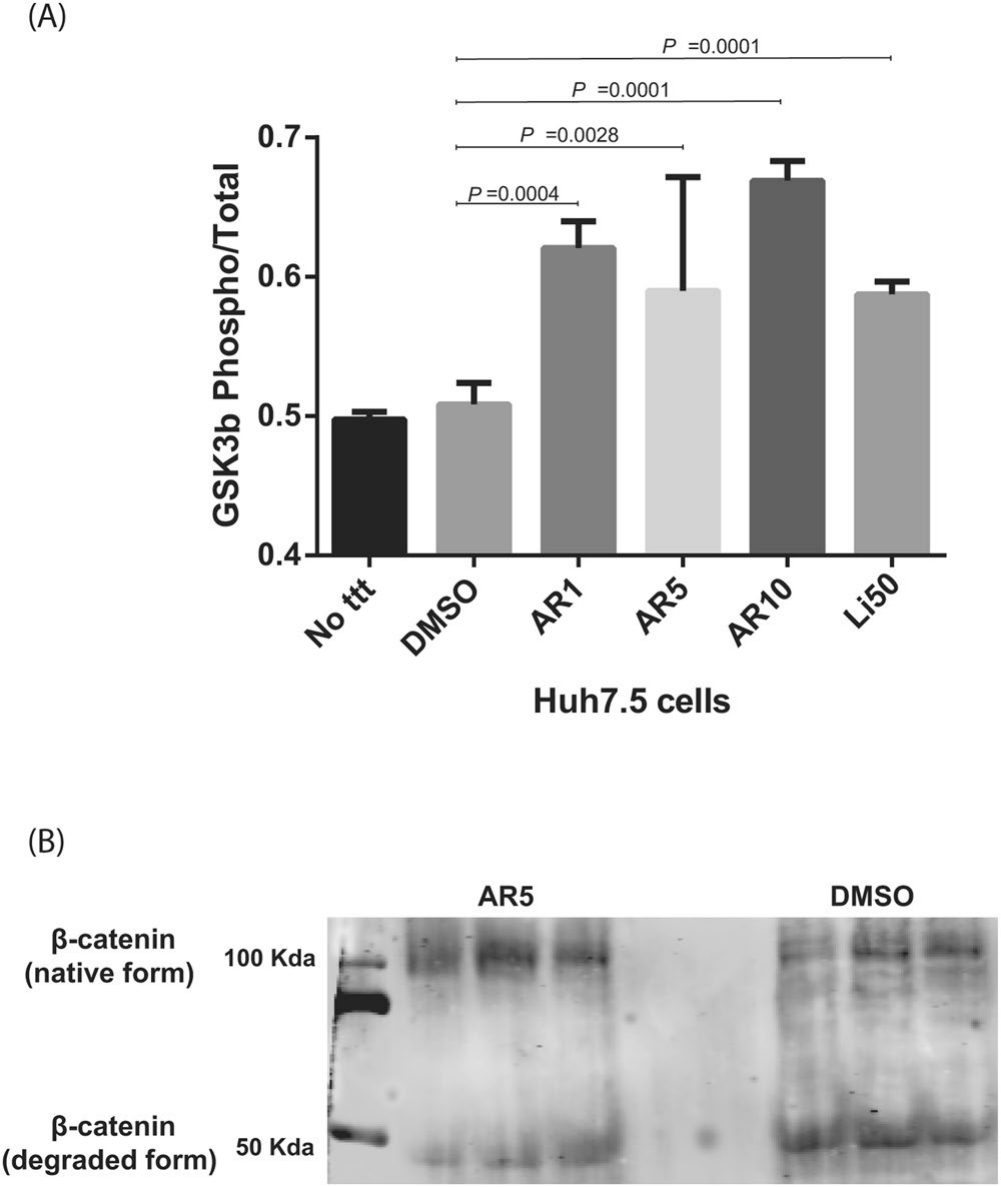
Increase in phosphorylated GSK3β and decrease in β-catenin degradation after treatment with GSK3 inhibitors. (**A**) Huh7.5 cells treated with AR1, AR5, AR10 and Li50 GSK3 inhibitors were harvested for protein. 25 µg protein was used to identify total and phosphorylated protein levels using ELISA. Values are expressed as the ratio of the phosphorylated to total GSK3β. Values = mean ± SD of three measurements in the same experiment. (**B**) Western blot analysis of Huh7.5 cell lysates for both the native and degraded form of β-catenin using specific mAb, shows that inhibition of GSK3β inhibits the degradation of β-catenin (n = 3 independent experiments). mAb, monoclonal antibody.

GSK3β is found constitutively in the active, non-phosphorylated form inside cells. Upon phosphorylation, GSK3β becomes inactivated. Li has a pleotropic effect on cells, whereas AR selectively inhibits GSK3β by competing for ATP binding with a Ki of 38 nM^18^. Following Li or AR treatment, we observed increased phosphorylated GSK3β relative to the total GSK3β. DMSO-treated or naïve cells were used as a control. Also, higher levels of pGSK3β was observed in AR- relative to Li-treated cells (Fig. 1A).

GSK3β together with APC, Axin, and CK1α form a destruction complex that mediates the hyperphosphorylation of β-catenin which is then subjected to ubiquitylation and degradation^7^. Thus, inhibition of GSK3β leads to stabilization and activation of β-catenin^19^. To demonstrate the effect of GSK3β on the expression of β-catenin, we extracted the protein from the same Huh7.5 cells treated before with AR5 for 24 hours in comparison to DMSO treated cells to identify the level of β-catenin by Western blot. An increase in the ratio of intact β-catenin (~100 kDa) to the degraded form was observed in AR5-treated cells (Fig. 1B). This finding confirmed the effect of GSK3β on β-catenin and is consistent with a previous study (9).

### GSK3β inhibitors have no effect on HCV replication

**Figure 2:**
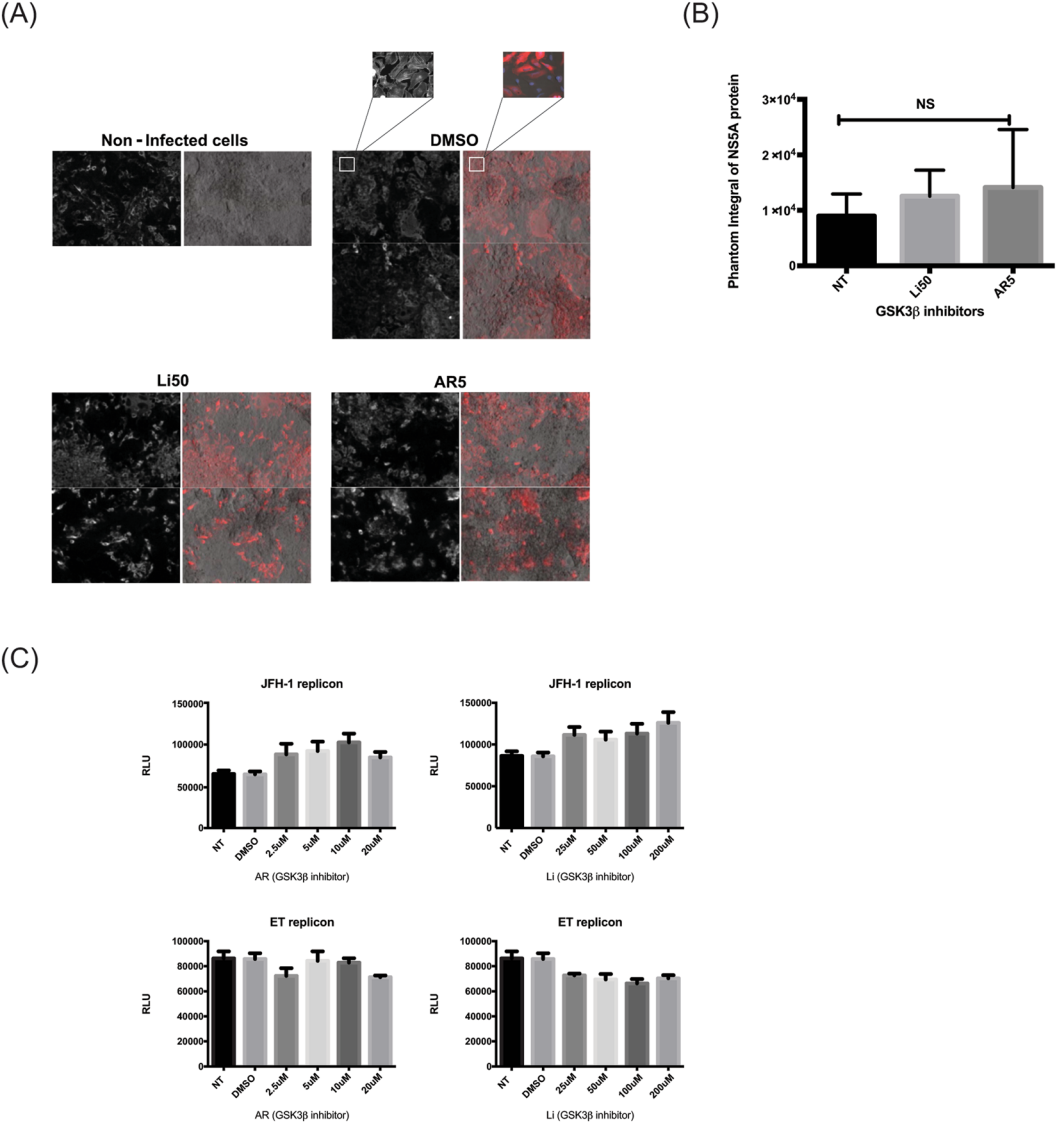
GSK3β inhibitors have no effect on HCV replication. NS5A expression was examined in Huh7.5 cells treated with DMSO or GSK3 inhibitors at 4 days post infection (dpi). Cells were treated with DMSO, Li50 or AR5 and infected with JFH-1 TCA. At 4dpi cells were fixed/permeabilized and stained with PE-labelled anti-NS5A mAb. (**A**) Laser scanning cytometry images of Huh7.5 cells treated with GSK3 inhibitors or DMSO. Transmitted light (Black and white, left panels) is used to show the density of the cells in each treatment. The red fluorescence is emitted by cells expressing NS5A protein (right panels). (**B**) The fluorescence quantified by the cytometer using the phantom integral mode of calculation is represented on the graph. (**C**) JFH-1 and ET, two replicon cells carrying HCV genotypes 2a and 1b, respectively, were seeded at a density of 1000 cells per well in 96 well plates and incubated at 37 °C. At 2dpi, the cells were treated with GSK3 inhibitors then lysed and washed for luciferase activity. HCV replication was reflected by luciferase activity using a commercial luciferase assay (Promega). Results are presented as means ± SD (n = 3 independent experiments). Dpi, days post infection, mAb, monoclonal antibodies, NS, non-significant.

As a first step to study the effect of GSK3β inhibitors on the HCV life cycle, we examined viral replication and NS5A protein expression in the Huh7.5 hepatoma cell line infected with HCV following treatment with GSK3β inhibitors and in two HCV genome-carrying replicon cells: replicon ET which is derived from the Con1 genotype 1b isolate and harbors adaptive mutations in NS3 and NS4B, and replicon JFH-1 that contains the genotype 2a JFH-1 isolate. Huh7.5 cells were pretreated for 24 h with lithium 50 µM (Li50) or AR 5 µM (AR5) and then exposed to JFH-1 for 4 h, washed 3 times, and re-cultured in fresh media. At 4 days post infection, cells were harvested for RNA and protein expression, or fixed in paraformaldehyde. Although immunohistochemistry revealed an increase in NS5A expression in cells treated with GSK3β inhibitors, no significant differences were observed in Li-, AR-, or DMSO-treated cells (Fig. 2A). Using the phantom integral mode of calculation, fluorescence was quantified (Fig. 2B). Further, HCV replication in genotype 2a and 1b replicon cells treated with GSK3β inhibitors was not affected after two days of treatment, as indicated by the absence of changes in luciferase activity in the cells (Fig. 2C).

### GSK3β inhibitors inhibited the release of HCV virions

**Figure 3:**
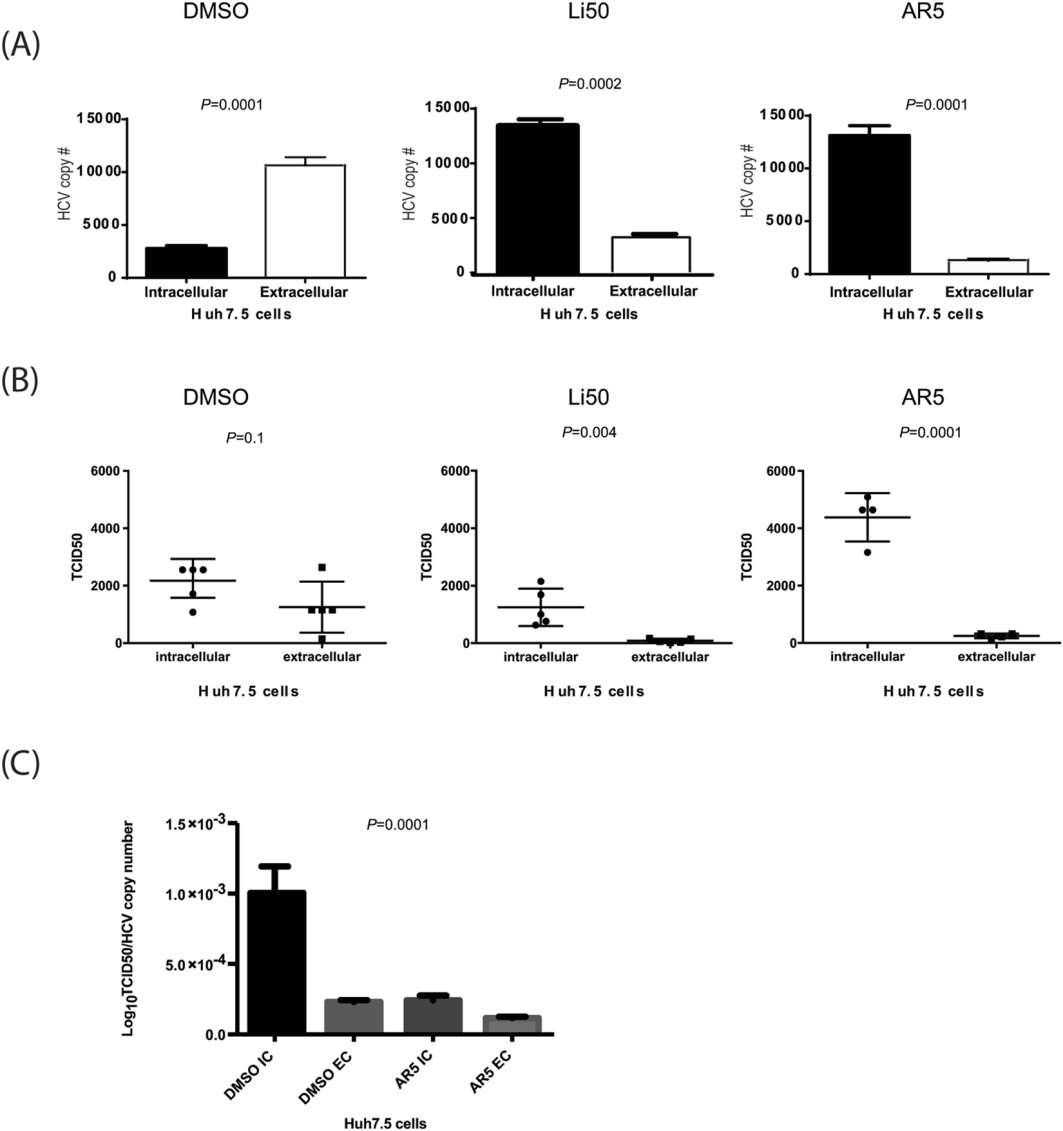
Decreased secretion of infectious HCV particles with GSK3β inhibitors. Decreased secretion of infectious HCV particles from Huh7.5 TCA cells treated with GSK3 inhibitor at 4dpi. IC and EC RNA were quantified using real-time RT-PCR (**A**). Cells were pre-incubated with either GSK3β inhibitor (Li and AR) or DMSO for 2 days then exposed to JFH-1TCA for 4 h. After 4dpi cells and medium were harvested for RNA extraction (**A**) and viral particles isolation (**B**). Intracellular RNA (**A**) but not TCID50 (**B**) was significantly higher in treated cells compared to the non-treated. Specific infectivity was much lower in treated cells IC and EC (**C**). Viral RNA titre was expressed as HCV copy number/ml, and infectivity of the virus as TCID50/ml. TCID50, tissue culture infective dose in 50% of the cells, IC, intracellular, EC, extracellular. The results are presented as the mean ± SD (n = 3 independent experiments).

RNA isolated from HCV-infected Huh7.5 cell culture was quantified by real-time RT-PCR for intra- and extracellular HCV RNA copy number (IC and EC, respectively). A large and significant decrease in HCV copy number in the supernatant of Li50- or AR5-treated cells was observed when compared to control, along with a significant increase within drug-treated cells (Fig. 3A). In addition, the infectious titer of the extracellular medium was significantly and dramatically reduced by drug treatment (Fig. 3B). The specific infectivity (calculated by dividing log TCID50 over the HCV copy number) of intracellular virus in AR5-treated cells was also clearly lower compared with control cells (to a lesser extent in extracellular virus from AR5-treated cells; Fig. 3C). Overall, these data indicate that GSK3β inhibitors affect the assembly and release of HCV from infected cells.

### Li and AR treatment were non-toxic to Huh7.5 and replicon cells

**Figure 4:**
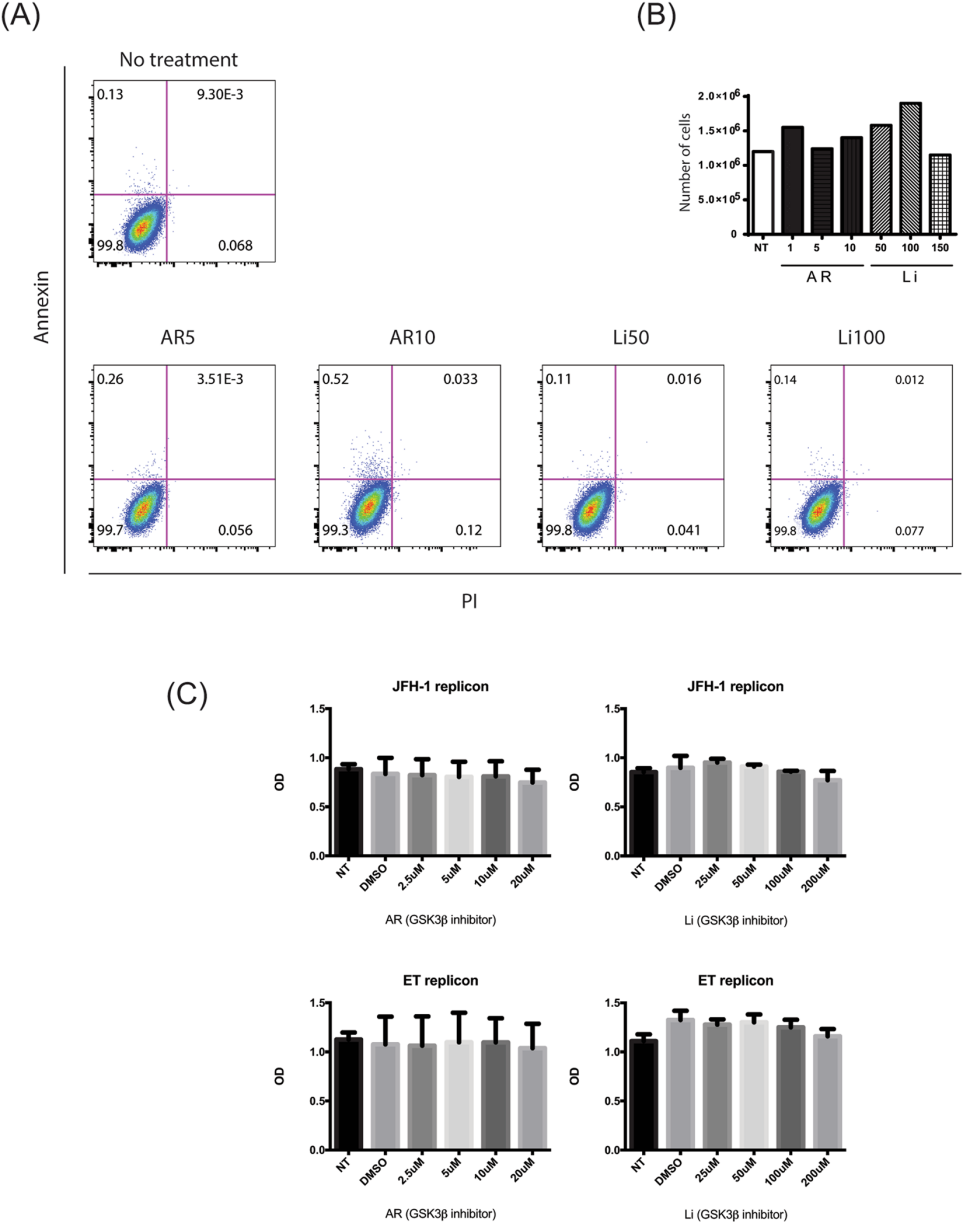
GSK3β inhibitors have no effect on cell apoptosis and cell death. (**A**) 10,000 cells were stained with annexin V and propidium iodide, and examined by flow cytometry. (**B**) No change in cell viability was also shown using trypan blue viability stain in treated and non-treated cells. (**C**) In parallel, cell proliferation and viability was demonstrated by the MTT proliferation assay on the used replicon cells. (n = 2 Independent experiments).

To exclude any effects of Li and AR on cell viability, and any subsequent effect on the amount of HCV produced, we treated cells with different concentrations of Li (25–200 µM) or AR (5–20 µM). Cell toxicity was assessed using Trypan blue staining, apoptosis markers, and the MTT cell proliferation assay (Fig. 4). In the Annexin V/propidium iodide apoptosis assay, cell viability exceeded 99.3% in all treated Huh7.5 cells (Fig. 4A). No significant differences were observed in treated versus non-treated cells at Li concentrations below 100 µM and AR concentrations below 20 µM in both the Trypan blue and MTT assays (Fig. 4B and C, respectively).

### GSK3β inhibitors did not affect other cell secretory functions

**Figure 5:**
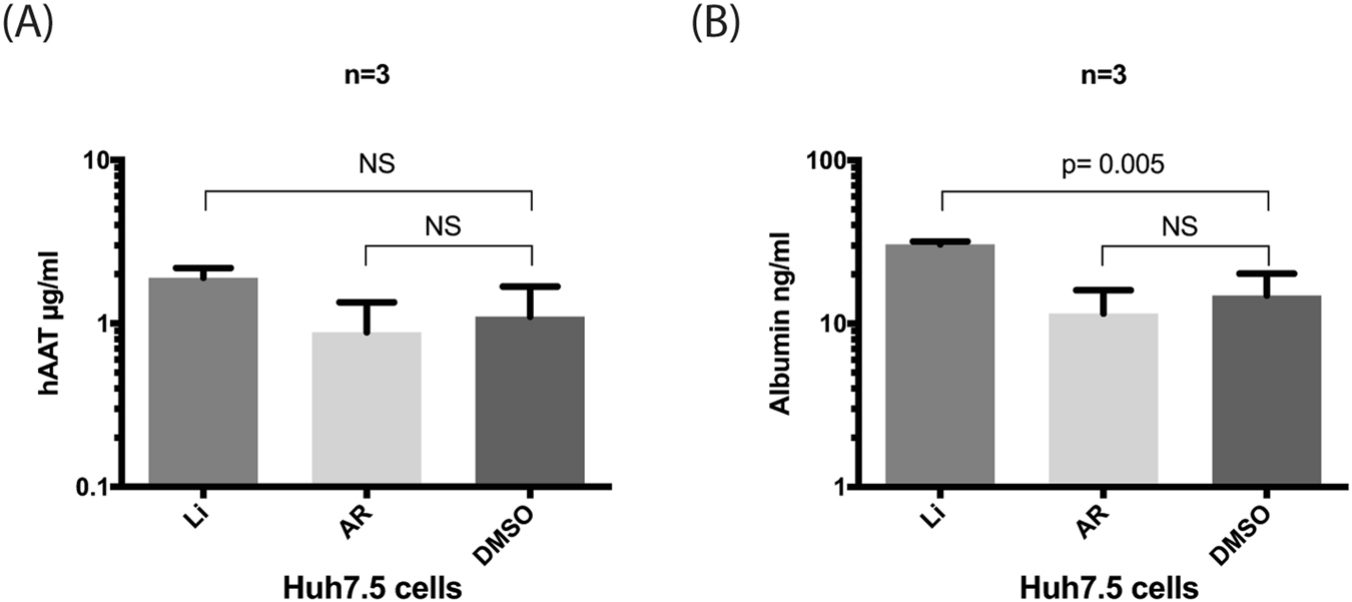
GSK3β inhibitors did not affect albumin or hAAT cell secretory functions. The levels of (**A**) hAAT and (**B**) Albumin were measured in the cell medium and show an intact secretory function of Huh7.5 cells before and after treatment. hAAT, human alpha1 anti-trypsin.

We examined the secretion of human alpha-1 antitrypsin (hAAT) and albumin using an ELISA (Fig. 5A and B, respectively). No significant changes in albumin and hAAT secretion were observed in Huh7.5 cells treated with AR5 in comparison to DMSO-treated cells. Apart from higher albumin secretion, Li50 has no effect on hAAT secretion. Thus, the effect of GSK3β inhibitors on HCV release was not due to inhibition of the overall cell secretory function.

### Reduction of triglyceride(TG)-rich fractions containing VLDL particles, ApoE, and ApoB in the supernatant of cells treated with GSK3β inhibitors

**Figure 6:**
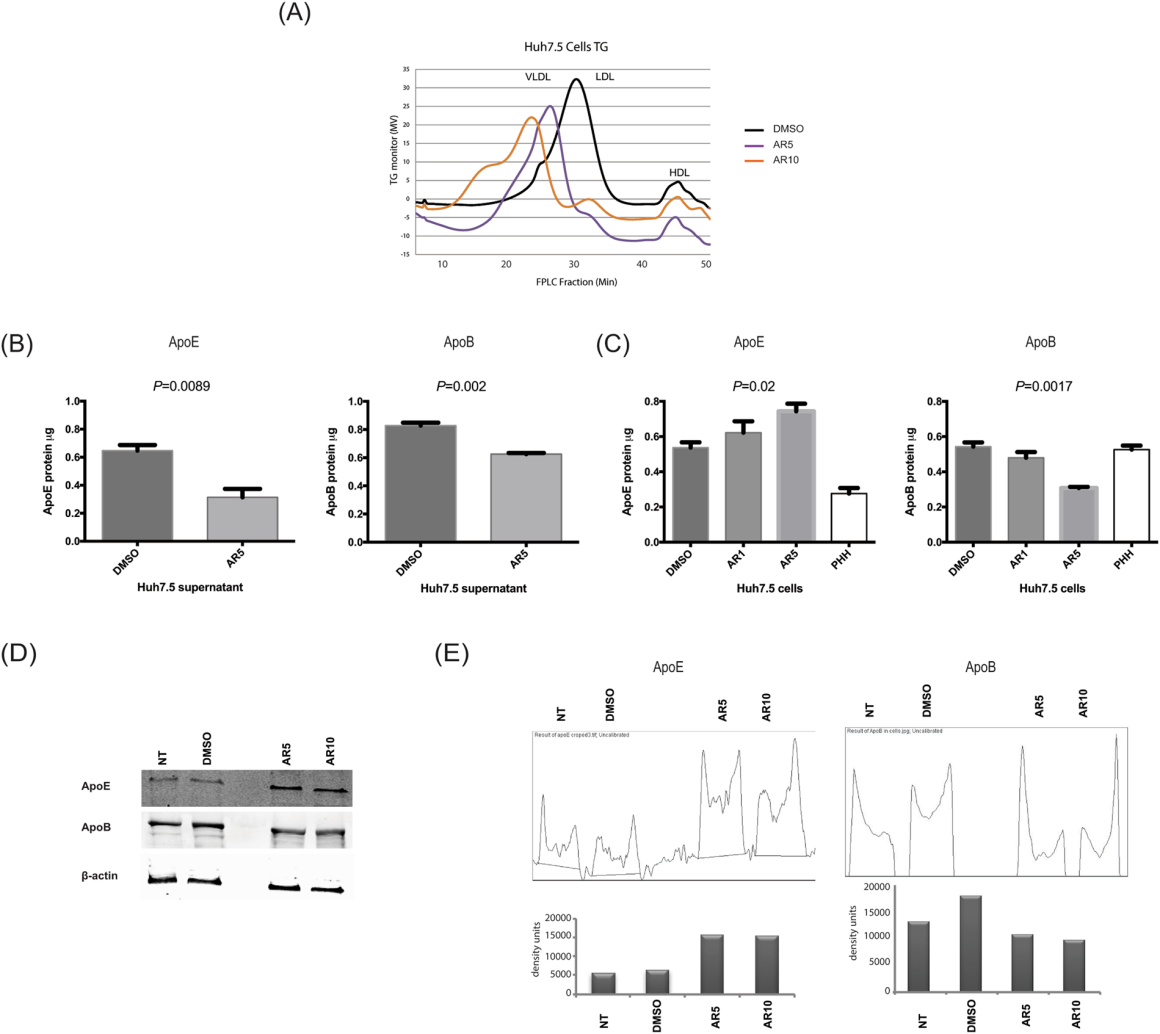
Decreased secretion of ApoE and synthesis of ApoB by Huh7.5 cells treated with GSK3β inhibitors. (**A**) FPLC shows dose dependent decrease in number and size of VLDL and LDL particles secreted by Huh7.5 cells treated with AR5 and AR10. (**B**) Decreased secretion of ApoE and ApoB protein by AR5-treated Huh7.5 cells. (**C**) A dose-dependent increase of ApoE and a decrease of ApoB synthesis inside the cells was noticed in AR1 and AR5 treated cells. PHH cells were used as a positive control for ApoE and ApoB protein expression and show a higher concentration of ApoB compared to ApoE. (**D**) ApoE and ApoB protein were detected in the same cells by western blotting on 5% SDS-polyacrylamide gels using specific mAb. The picture was cropped from the actual blot. (**E**) Densitometry plots for quantification of the bands in the western blot in panel (**D**), confirm the increase in intracellular ApoE (left panels) and decrease in intracellular ApoE. FPLC, fast protein liquid chromatography, mAb, monoclonal antibodies, PHH, primary human hepatocytes, VLDL, very low density lipoprotein. The results are presented as the mean ± SD (n = 3 independent experiments).

The maturation and production of HCV virions *in vivo* is dependent on the pathway of VLDL assembly and release^20^. This process requires ApoE and ApoB which are an integral part of the HCV lipo-viral particle (LVP)^20^. In Huh7.5 cells, despite the deficient production of VLDL, virus maturation depends mainly on ApoE^21^. Additionally, GSK3 phosphorylates some transcription factors that are implicated in lipid metabolism^22^. Thus, we measured the levels of triglycerides-rich fractions secreted by treated Huh7.5 cells (Fig. 6A). Concentrated cell supernatant was used to identify lipid secretions in treated cells and controls. The number and size of the TG-rich particles including VLDL was downregulated in cells treated with AR5 and AR10 compared to DMSO (Fig. 6A). We then tested the levels of ApoE and ApoB in the supernatant and cells using ELISA techniques (Fig. 6B and C, respectively). Primary human hepatocytes served as a positive control for ApoE and ApoB (Fig. 6C). Although, the levels of both ApoE and ApoB were significantly attenuated in the cellular supernatant, intracellular ApoE was enriched compared to ApoB which was significantly downregulated (Fig. 6C,D and E). The perturbation in lipoprotein biosynthesis due to GSK3β inhibitors may impact HCV maturation and release by Huh7.5 cells. Our findings are partly consistent with findings by Change and colleagues who demonstrated that inhibition of GSK3β decreased the number and size of VLDL particles due to an effect on ACL3, which inhibited the upregulation of lipids induced by ER stress^23^.

### GSK3β inhibitors had no effect on lipid droplets but were associated with the accumulation of inclusion bodies inside the cytoplasm

**Figure 7:**
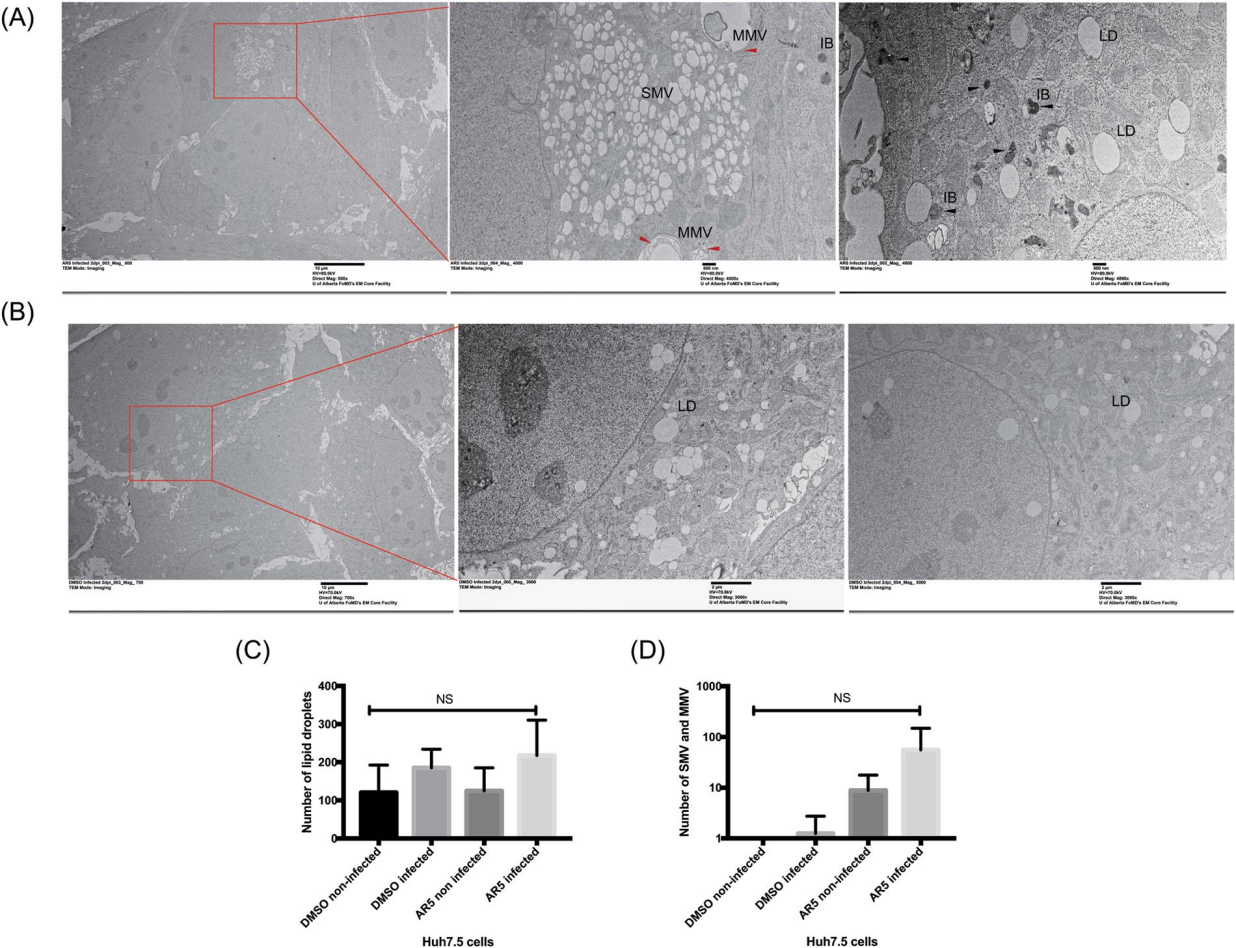
Cells treated with GSK3β inhibitors accumulate single and multivesicular bodies and tend to have more inclusion bodies. EM analysis of fixed Huh7.5. Cells cultured on thermanox slides. After 4dpi sections were prepared for EM imaging. (**A**) Cells treated with GSK3β inhibitors show accumulation of single and multivesicular bodies (middle panel, red arrows) which was also apparent by counting the number of SMV and MMV (**D**). Accumulation of inclusion bodies was observed (shown by the black arrows, right panel). This was not obvious in sections taken from control cells treated with DMSO (**A**,**B**). Lipid droplets were enriched in virus infected cells as shown by the number of lipid droplets counted from 10 fields (**C**). MMV, multiple-membrane vesicles, SMV, single membrane vesicles.

It has been reported that HCV can induce the accumulation of lipid droplets, possibly through the expression of core proteins^13^. We also observed that lipid droplets accumulated in HCV infected cells as recorded by the average number of lipid droplets observed per cell in 3 different sections examined by electron microscopy. The lipid droplets accumulated only in HCV-infected Huh7.5 cells and no significant difference was observed in AR5- versus DMSO-treated cells (Fig. 7C). Nevertheless, more single and multi-membrane vesicles were observed in cells treated with GSK3β inhibitors (Fig. 7A, red arrows and 7D) compared to DMSO-treated cells (Fig. 7B). In certain fields increased formation of inclusion bodies was apparent in AR5-treated cells (Fig. 7A, black arrows) in comparison to DMSO-treated cells (Fig. 7B). This may reflect attenuated autophagy mediated by GSK3β inhibition as reported by others^24, 25^. Non-infected and treated Huh7.5 cells showed no accumulation of lipid droplets, which indicates an effect only in HCV-infected cells.

## Discussion

Although many models have been proposed for HCV assembly and release from cells, the actual mechanism remains to be further defined. The classical link between HCV lipo-viral particles and the association with VLDL assembly and release has been shown previously^21^. However, new pathways including the canonical endoplasmic reticulum-Golgi intermediate compartment, as well as the endosome-dependent pathway have been shown to be involved in HCV particle release^26, 27^. Hence, growing evidence supports the involvement of host proteins in viral entry, assembly, and release, and emphasizes the importance of targeting host proteins as a broad spectrum antiviral approach. In this study, we emphasized the importance of host proteins on HCV virion maturation and release. We identified GSK3β as a key molecule in HCV virion maturation and release and is associated with alteration of the lipid biosynthesis. We also, demonstrate GSK3β as a therapeutic target for HCV treatment.

Based on unpublished clinical observation that shows patients with chronic HCV, who are on Li treatment for bipolar disorders, tend to have better liver function profile and improved general condition, and based on the role of lipids in HCV assembly and/or release, our primary aim was to investigate the effect of GSK3β inhibitors, such as Li and AR on HCV replication, virus assembly and release.

First, we confirmed that Li or AR enhanced the phosphorylation of GSK3β and inhibit the molecule (Fig. 1A). The inactivated GSK3β was associated with a less degraded β-catenin (Fig. 1B) which confirm that GSK3β molecules were inhibited by our drugs. Interestingly, no effect on HCV replication in Huh7.5 cells or replicon cells after treatment with Li or AR (Fig. 2A and C). This was evident by no effect on viral NS5A protein expression (Fig. 2A) in Huh7,5 cells infected with JFH-1 and by the luciferase activity in two replicon cells with different genotypes. More importantly, Huh7.5 cells treated with GSK3β inhibitors produced less HCV viral particles in the cell supernatant (Fig. 3A). These findings suggest an effect on HCV at later stages of assembly and/or secretion rather than the earlier stages. The effect on release was evident by lower HCV copy numbers in treated cells compared to the control. Also, isolating the intracellular HCV viral particles by repeated freeze thaw and calculating the TCID50 for both the IC and EC compartments shows and effect on the specific infectivity which indicate that GSK3β may affect the assembly of HCV. To further understand the mechanism of GSK3β on the maturation of HCV, we examined the effect on lipids by FPLC that shows a total shift of the TG-rich fraction towards having lower number and size of VLDL and LDL molecules. Although the shift also reflects having lower density, infectivity was not improved, on the contrary, a lower number of virions was released with decreased specific infectivity. This strongly suggests that other factors related to inhibition of GSK3β could be the reason behind the decrease in released virions. Therefore, we investigated the effect of GSK3β on apolipoproteins E and B, taking into consideration the proven role of ApoE in the release of mature HCV viral particles in Huh7.5 cells^20, 28^. Interestingly, inhibition of GSK3β inhibited the synthesis of ApoB and appeared to trap ApoE molecules inside the cells (Fig. 6B,C), which may explain the suppressed release of HCV virions (Fig. 3). The inhibition of GSK3β apparently affected both virus assembly and release, since there is a reduction in the specific infectivity of intra-cellular virions in cells treated with GSK3β inhibitors compared to DMSO treated cells, in addition to the significant decrease in viral RNA and infectious viruses secreted into the cell supernatant. These results suggest a GSK3β effect on late stages of virus release/assembly which could be attributed to inhibition of apolipoproteins secretion.

GSK3β is also a member of the CMCG phosphorylation family that phosphorylates serine residues in hundreds of molecules^29^. The HCV NS5A protein contains several phosphorylation sequence motifs, and the phospho amino acid analysis of NS5A of HCV genotype 1a indicated that NS5A phosphorylation occurs predominantly to serine residues, with a minor fraction of threonine residues also being phosphorylated^30^. The link between both observation is yet to be clarified and further studies are needed.

Another interesting observation, is the increase in the number of inclusion bodies in the cytoplasm of GSK3β inhibitor-treated cells. This could be attributed to the accumulation of viral proteins or lipoviral particles in cellular vacuoles (Fig. 7). In addition to their proposed antiviral effects, GSK3β inhibitors were shown to be protective against acute liver failure. This adds to the benefits of using these molecules in patients with advanced liver diseases^31^. Nevertheless, little data is available on the side effects of GSK3β inhibitors, due to the limited number of compounds that reached the clinical phase.

**Figure 8:**
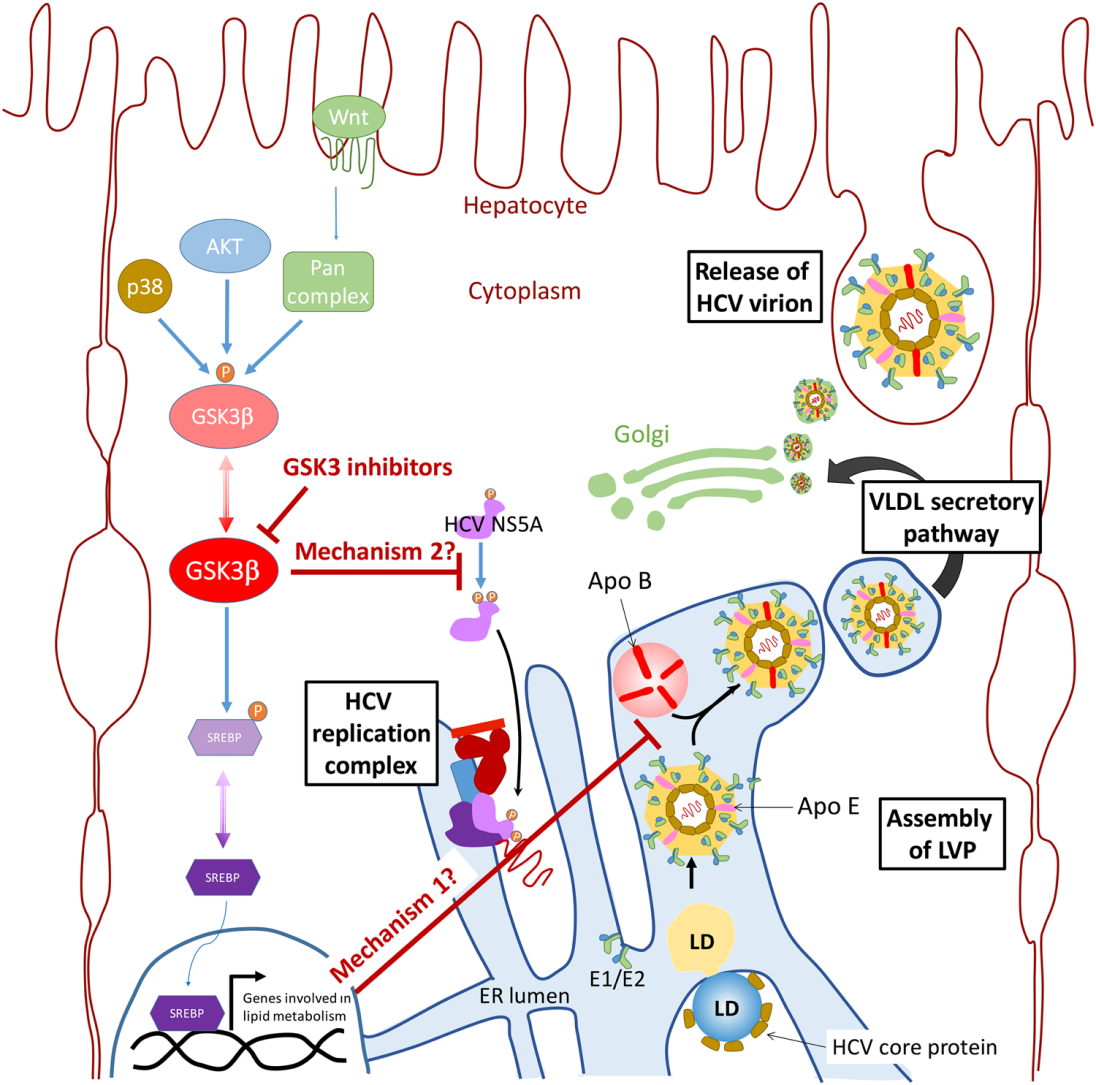
Proposed model for the effects of GSK3β inhibitors on HCV virion particles’ assembly/release. GSK3β is an important kinase that is capable of phosphorylating a few hundreds of proteins, including SREBP-1 and acyl-CoA synthetase 3 (ACSL3) which are involved in lipid metabolic pathways^23, 32^. The phosphorylated (inactive) form is downstream the canonical Wnt pathway, which is stimulated by various signals/stimuli, as well as the p38 protein and AKT^33^. The adherence of HCV virion assembly to the host cell’s lipid metabolism and secretory pathways renders it sensitive to lipid perturbations^34^. The effect of GSK3β enzymes and transcription factors involved in lipid metabolism hinders the assembly/release of HCV LVPs (potential mechanism 1). GSK3b is also a member of the cyclin-dependent kinase CMGC family. Members of this family were shown to be involved in the phosphorylation of HCV non-structural protein 5A (NS5A), which is an integral part of the HCV replication complex^31^. Similarly, GSK3β inhibitors may interfere with this process, representing another potential mechanism for inhibition of HCV (potential mechanism 2). *The diagram is a schematic drawing and is not proportionate to the real dimensions.

In conclusion, targeting host cell proteins such as GSK3β may inhibit the assembly/release of HCV viral particles, and is associated with inhibition of apolipoprotein E and B release. The results provide an evidence for the antiviral effect of GSK3β inhibition and offer ﻿a new therapeutic modality in the treatment of HCV. It also adds new insights to our understanding of the HCV biology. A proposed model that highlights potential mechanisms of action for GSK3β inhibitors, and the possible effect on later stages of HCV maturation and release is depicted in Fig. 8.
